# Highlighting the Relevance of CD8^+^ T Cells in Filarial Infections

**DOI:** 10.3389/fimmu.2021.714052

**Published:** 2021-09-16

**Authors:** Alexander Kwarteng, Ebenezer Asiedu, Kelvin Kwaku Koranteng, Samuel Opoku Asiedu

**Affiliations:** ^1^Department of Biochemistry and Biotechnology, Kwame Nkrumah University of Science and Technology (KNUST), Kumasi, Ghana; ^2^Kumasi Centre for Collaborative Research in Tropical Medicine (KCCR), Kumasi, Ghana; ^3^Department of Theoretical and Applied Biology, Kwame Nkrumah University of Science and Technology (KNUST), Kumasi, Ghana

**Keywords:** anti-filarial immunity, T cell, CD8^+^, filarial co-infections, filarial pathology

## Abstract

The T cell immune responses in filarial infections are primarily mediated by CD4^+^ T cells and type 2-associated cytokines. Emerging evidence indicates that CD8^+^ T cell responses are important for anti-filarial immunity, however, could be suppressed in co-infections. This review summarizes what we know so far about the activities of CD8^+^ T cell responses in filarial infections, co-infections, and the associations with the development of filarial pathologies.

## Filarial Infections: An Overview

**Figure 1:**
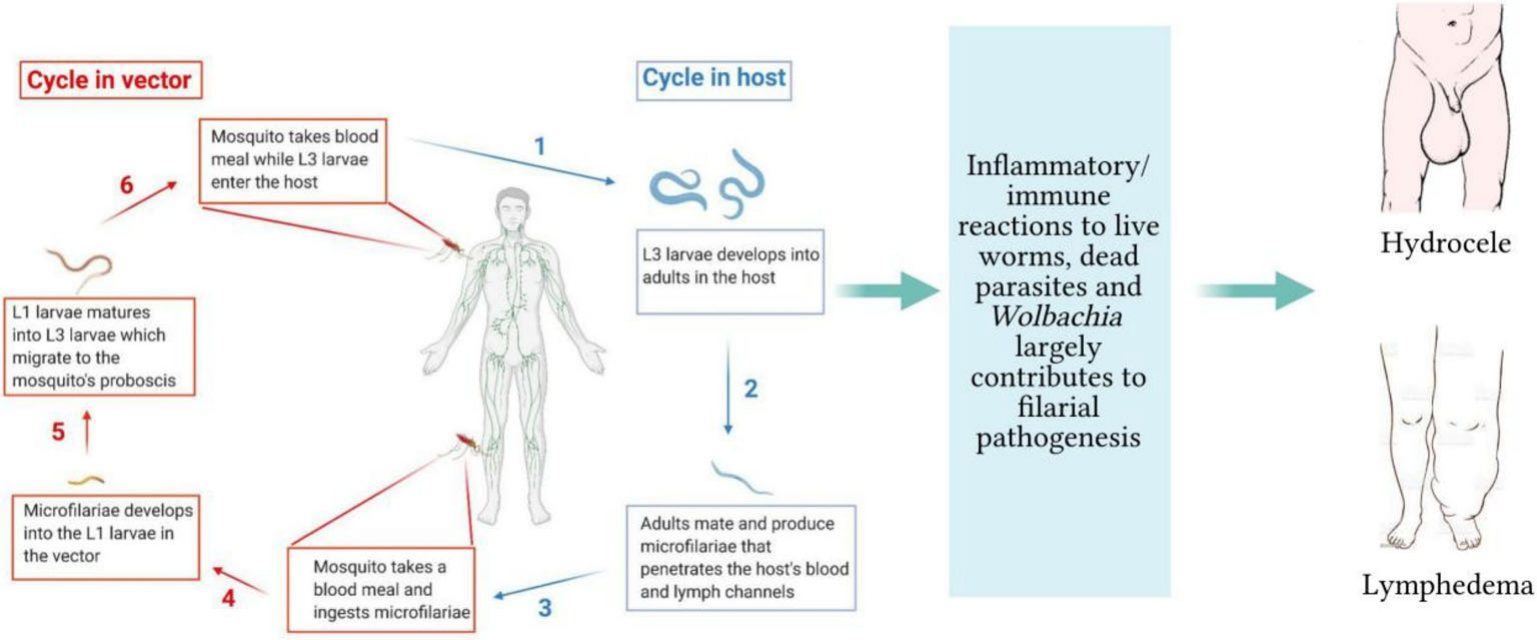
Life-cycle of filarial parasites demonstrated with W. bancrofti and the associated filarial pathologies. (1) The vector transmits the infective-stage larvae into the human when taking a blood meal. (2) The L3 mature into adult worms, which produce microfilariae (3). They migrate to the lymphatics and blood for circulation. (4) The vector again takes up the microfilariae during a blood meal on an infected host. (5) The microfilariae develop into the L1 stage. (6) The L1 larvae mature into L3 larvae, which migrate to the vector’s proboscis *via* the haemocoel. (1) The infected vector transmits the infective-stage larvae into the human host during a blood meal. While in the host, inflammatory responses to live/dead parasites and immuno-regulatory mechanisms contribute to lymphedema and hydrocele.

Filarial infections of animals and humans include lymphatic filariasis, onchocerciasis, loiasis, and mansonellosis. Lymphatic filariasis is caused by *Wuchereria bancrofti*, *Brugia malayi*, and *Brugia timori*, with *W. bancrofti* accounting for over 90% of such infections (1). The *Onchocerca volvulus* is responsible for onchocerciasis, popularly called river blindness. The other disease-causing species of filarial parasites are *Loa loa* and *Mansonella* sp., causing loiasis and mansonellosis, respectively. The life-cycle of filarial parasites is relatively complex with several distinct morphological stages in both vector and mammalian hosts, as shown with *W. bancrofti* in **Figure 1**. For lymphatic filariasis (LF), the most prominent pathological manifestations are mediated by immune responses against the adult worms and infective stage larvae (2), leading to lymphedema and hydrocele (**Figure 1**). LF is the second largest cause of disability globally and approximately 40% of the global disease burden of lymphatic filariasis occurs in Africa (3). Current treatment strategies include mass drug administration (MDA) regimens and vector control measures. The MDA programs involve the yearly distribution of microfilaricidal drugs constituting single doses of 400 mg of albendazole (ALB) plus either 150–200 mg/kg of ivermectin (IVM) or 6 mg/kg of Diethylcarbamazine (DEC) administered together for 4–6 years. Tetracycline-based drugs such as doxycycline are used as macrofilaricidal agents (4, 5). Vector control strategies have been used to effectively interrupt the transmission of LF in certain countries (6).

## Helper T Cells in Anti-Filarial Immunity and Immunoregulation: An Overview

T cell-mediated immune responses are major components of the anti-filarial immunity, as per evidence from experimental animal models and antigen stimulation studies (7–9). This T cell-mediated response has been characterized by activities of both T helper 1 and T helper 2 cytokines, depending on the stage of parasite/infection (10–12). At the interplay of the Th1/Th2 activities are regulatory T cells (Treg) which may be simultaneously active during the filarial infections (13, 14). Thus, several scenarios of helper T cell responses could be observed. First, the responses can be dominated by strong Th1 cytokines with diminished Th2 cytokine activity as a result of poor regulation by Treg. Another scenario could involve a dominant type 2 cytokine response with a marginal activity of Th1, due to immunoregulatory activities. There could also be well-balanced Th1/Th2 responses when Treg activities are sufficient. The activities of primed CD4^+^ T cells involved in *Onchocerca* microfilariae clearance in infected mice are dominated by Th2 responses (15). In the early stages of filarial infection, an increased expression of Th1 cytokines, particularly triggered by antigen-presenting cells have been reported (7). This suggests that the initial responses against infective stage larva involve pro-inflammatory responses induced by innate components although the primary responses against the parasites are of Th2 phenotype. A large body of evidence shows that the interactions with host innate cells involve toll-like receptors (16–18). The *Wolbachia* induces dendritic cell activation and IFN-γ secretion which are correlated with increased TLR2 expression (19). Studies with *O. volvulus* keratitis-infected mice demonstrated that IFN-γ increased expression of TLR2 on corneal macrophages which triggered the production of TNF-α, interleukin-6 (IL-6), IL-1α, and IL-1β in macrophages (17). Moreover, TLR2 is involved in dendritic cell activation, IFN-γ secretion, and neutrophil recruitment (17, 18). In addition to TLR2, the *Wolbachia* induces innate immune responses through TLR4 and TLR6 and involves MyD88 (18, 20). The *W. bancroft*i microfilaria sheath protein directly interacts with TLR4 to mediate macrophage pro-inflammatory responses (IL-6, TNF-α, and IL-1β) *via* NF-kB activation (20, 21).

**Figure 2:**
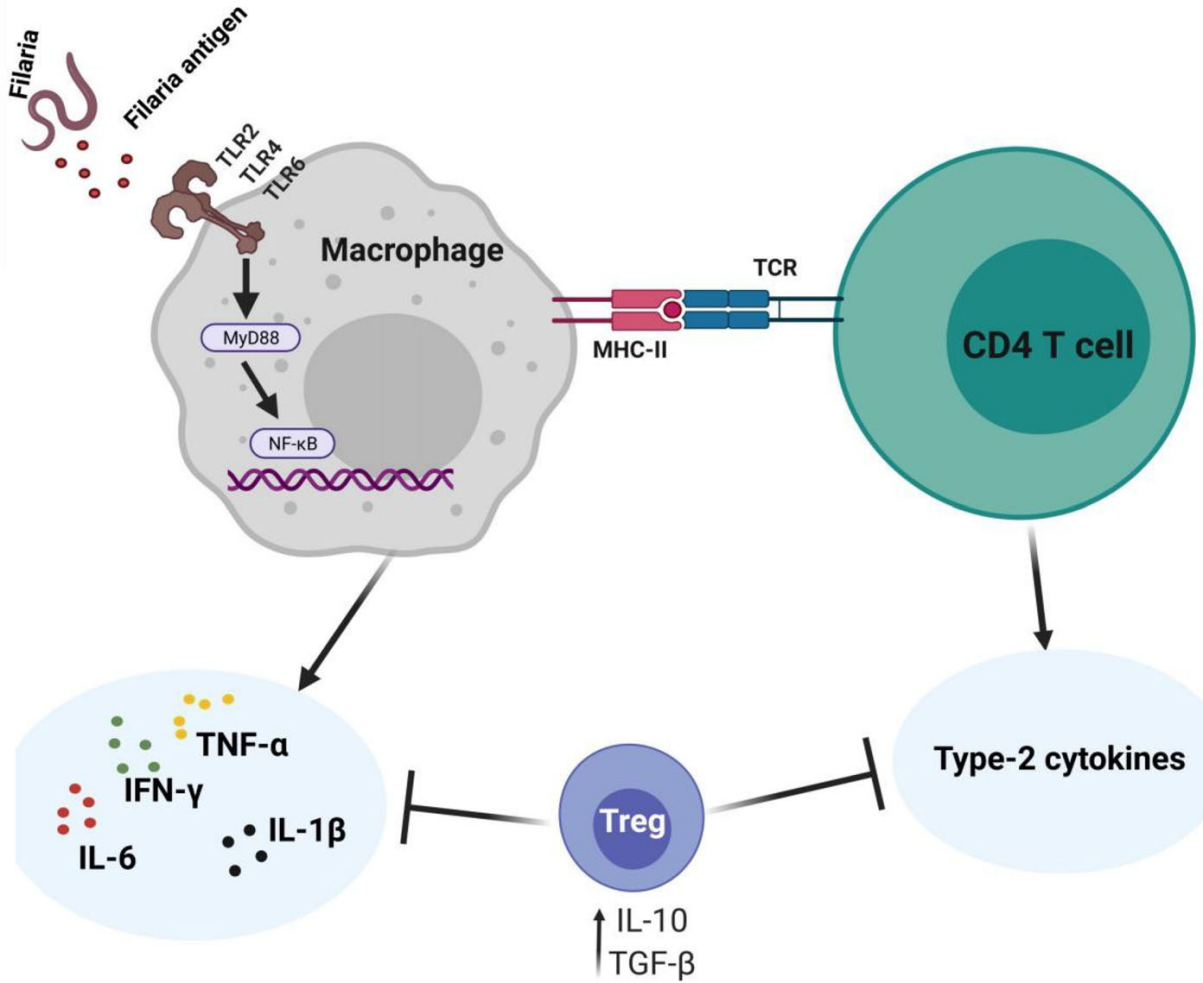
Cytokine expressing profiles against filaria and immunoregulation. The filaria and their endosymbiont interact with TLRs on the APCs to induce pro-inflammatory responses *via* NF-kB signaling. The CD4^+^ T cells expressing type-2 cytokines are involved in parasite clearance. Regulatory T cells are simultaneously involved in modulating the Th1/Th2 responses.

Reports by Mukherjee et al. (22) demonstrated that *W. bancrofti* sheath antigen promotes maturation and activation of dendritic cells by directly interacting with TLR4. In the same study, the matured dendritic cells promoted type-1 cytokines and regulatory T cells (Treg) responses while proportions of Th2, Th17, IL-4, and IL-17A were low (22). While the low Th2 responses could be a characteristic of the sheath antigen, there is a possibility of immunoregulatory activities by Treg. Studies have shown that the Th2 responses to *B. malayi* adult worm antigen are enhanced upon abrogation of Treg (23). Elsewhere, depletion of Treg promoted Th1 responses without affecting Th2 proportions (24). The *Wolbachia* surface protein promotes pro-inflammatory responses in mice by increasing the production of Th17 cells while decreasing the levels of Treg (25, 26). In addition, neutralizing CD25 and GITR in *B. malayi* L3-infected mice elevated Th17 and IFN-γ levels while reducing IL-10 (26). **Figure 2** summarizes the mediation of cytokine responses through antigen-presenting cells during filarial infections. This interplay of the type-1 and type-2 CD4^+^ T cell responses in anti-filarial immunity and the immunomodulatory actions of Treg have been extensively reviewed elsewhere (13, 14).

Here, we discuss the relevance of CD8^+^ T cells in the immune responses against filarial parasites, based on documented evidence from animal models and human subjects. We also discuss the roles of CD8^+^ T cells in the development of filarial pathologies and their possible involvement in immunomodulatory mechanisms during filarial co-infections.

## CD8^+^ T Cells in Anti-Filarial Immunity

### Evidence From Studies Involving Humans

There is currently insufficient evidence from human studies which clarify the mechanisms of CD8^+^ T cell activities in anti-filarial immunity. However, available data from studies in human subjects demonstrate the elevated levels of CD8^+^ T cells during filarial infections, thus, depicts potential roles in the immune responses against filarial parasites or immunopathology. Kalinkovich and colleagues examined a group of helminth-infected Ethiopian immigrants in Israel and reported elevated CD8^+^ T cells in this infected group (27). Moreover, *W. bancrofti*-infected individuals reportedly had increased frequencies of activated CD8^+^ T cells compared to non-infected subjects (28). From a cohort of 12 African adults with ocular onchocerciasis, Chan et al. found that levels of CD8^+^ T cells were significantly elevated in the infected group (29). There are additional reports that CD8^+^ T cells may have regulatory roles during the induction of ocular inflammation by *O. volvulus* antigen-specific CD4^+^ T cells (30).

**Figure 3:**
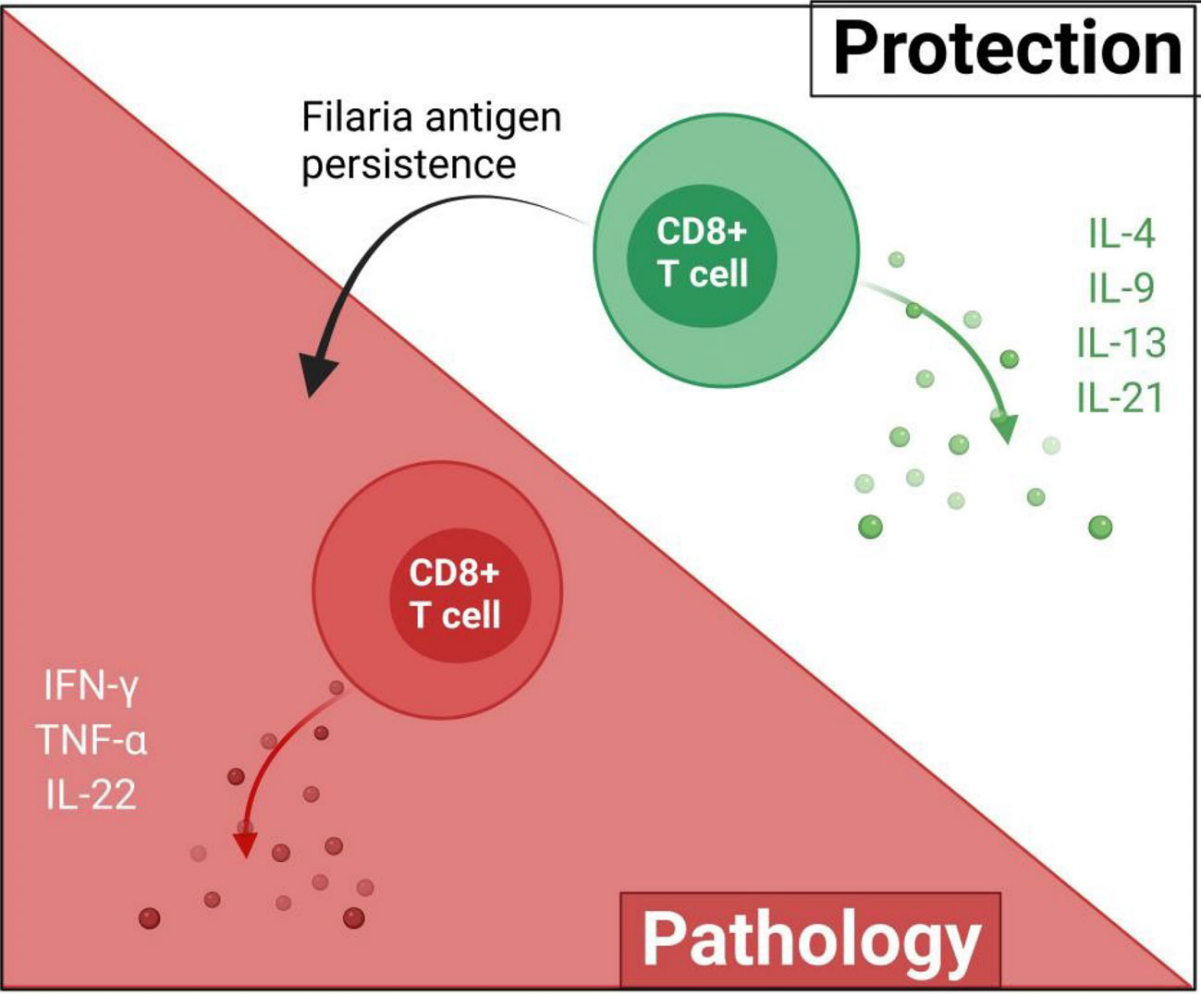
The possible roles of CD8^+^ in filarial immunity. The protective effects of CD8^+^ T cells are mediated by type-2 cytokines and upon the persistence of the filaria antigens, switch to pro-inflammatory type-1 responses which ultimately contribute to filarial pathology.

Filarial antigen stimulation studies suggest that type 2 cytokines mediate the cytotoxic activities of CD8^+^ T cells against filarial parasites. In individuals with patent infections, CD8^+^ T cells expressing antigen-specific type 1 cytokines (IFN-γ, TNF-α, and IL-22) were diminished, while levels of type 2 cytokine (IL-4, IL-9, IL-13, and IL-21)-expressing CD8^+^ T cells were elevated (31). This may suggest that CD8^+^ T cell responses in patent LF infections are mediated by antigen-specific type 2 cytokines (31, 32). Notwithstanding, treatment of the filarial infection changes the CD8^+^ T cell cytokine secretion pattern, thus, the regulation of these patterns of CD8^+^ T cell cytokine release is dependent on antigen presence or disease state (31, 32). The activities of CD8^+^ are modulated by IL-10 depending on the stage of infection (31, 32). CD8^+^ T cells expressing IL-24 and IL-19 were increased while IL-26 expressing CD4^+^ T cells were diminished in asymptomatic infected individuals compared to individuals presenting with pathology (32). These regulation of parasite antigen-specific IL-19 and IL-24 expressing CD8^+^ T cells are more dependent on the stage of the infection than on IL-10, IL-1β, and IL-23 as observed in the regulation of CD4^+^ T cell response (31–33). Filaria antigen-specific type-1 cytokines (TNF-α, IFN-γ, IL-22) expressing CD8^+^ T cells were reportedly lower in asymptomatic infected individuals, relative to individuals presenting with pathology (31). This evidence supports the notion that CD8^+^ T cell responses in filarial infections may be mediated by type 2 cytokines while filarial pathologies are driven by type 2 responses, as a result of continuous antigen presence and immunomodulation (**Figure 3**).

### Evidence From Studies With Animal Models

An early description of T cell-mediated immunity in experimental filaria excluded contributions from CD8^+^ T cells (34). β_2_-microglobulin deficient mice, which lack MHC class I molecules and therefore do not elicit CD8^+^ T cell cytotoxicity, were able to establish a resistance to the infective parasites of *B. malayi* (34). Several *in vivo* immunization studies, however, also suggested possible CD8^+^ T cell cytotoxic activities during filarial infections. The *B. malayi* trehalose-6-phosphate phosphatase antigen induced high levels of CD8^+^ T cells as part of the host antigen-specific cellular immune responses (35). Immunization of mice with *B. malayi Wolbachia* recombinase A resulted in a significant expansion of CD8^+^ T cells in the host spleen (36). Studies in TCR transgenic mice have also shown that the *O. volvulus* antigens O*v*ALT-2 and O*v*NLT-1 exert suppressive effects on CD8^+^ T cell proliferation and cytokine release (37). Recently, heterologous prime-boost vaccination administration of *B. malayi* heavy chain myosin induced enhanced CD8^+^ T cell expansion in mouse spleen (38). Although CD8^+^ T cell levels are elevated during filarial infections, it may be that CD8^+^ T cell-mediated immunity is most relevant at the initial stages of the infections when the parasite is establishing itself in the host. Babu and Nutman explored the events of host immunity that occur in the early stages following infection with live *B. malayi* L3 parasites and reported that efficient CD8^+^ T cell responses are noticed at the very early stages of infection (7). Studies with *Onchocerca lienalis*-infected mice model demonstrated the contributions of T cell-mediated immunity against microfilariae, which is dominated by CD8^+^ T cells at the initial stages of the infection (15). The filaria-specific CD8^+^ T cells expressing cytokines during filarial infections are summarized in **Table 1**.

**Table 1:** CD8^+^ T cells expressing cytokines in response to filaria infections.

Main Finding	Study model	Experimental design	CD8^+^ expressing cytokines	Ref
*Onchocerca volvulus* antigens (*Ov*ALT-2 or *Ov*NLT-1 displayed) suppressed CD8^+^ T cells-derived IL-2 and IFN-γ in mice	Mouse	*In vitro* proliferation assay using TCR transgenic mice	IL-2 and IFN-γ	(37)
*L. sigmodontis* infection suppressed the function of CSP-specific CD8^+^ T cells	Mouse	immunization with a circumsporozoite protein (CSP) fusion protein in *L. sigmodontis*-infected mice	IFN-γ and TNF-α	(39)
*B. malayi* L3 stage induced CD8^+^ T cell-associated Th1 cytokines	Cell culture	*in vitro* system of PBMC from unexposed individuals stimulated with live L3 of *B. malayi*	IFN-γ and TNF-α, GM-CSF, IL-1α, and IL-8	(7)
Infected asymptomatic individuals have elevated frequencies of CD8^+^ T cells expressing IL-4, IL-5, IL-9, IL-13, and IL-21 compared to individuals with clinical pathologies but lower type-1 cytokine expressing CD8^+^ T cells	Cell culture	*B. malayi* antigen stimulation of whole blood from infected individuals	Infected asymptomatic infections:	(31)
			IL-4, IL-5, IL-9, IL-13, and IL-21	
			clinical pathology:	
			IFN-γ, TNF-α, IL-22	
CD8^+^ T cells expressing IL-19 and IL-24 are characteristic of asymptomatic infections while IL-26 expressing CD8^+^ T cells are associated with the clinical pathologies	Cell culture	*B. malayi* antigen stimulation of PBMC from infected individuals	asymptomatic infections:	(32)
			IL-19, IL-24	
			clinical pathology:	
			IL-26	

## CD8^+^ T Cells and Associations With Filarial Pathology

The degenerating filaria and its endosymbiont promotes pro-inflammatory responses which drive the pathologies associated with the disease. The interactions between the microfilariae (Mf) and host macrophages result in the activation of pro-inflammatory macrophage subtype which induces a pro-inflammatory response (22, 40) aimed at promoting microfilariae clearance (7, 41). However, the macrophage pro-inflammatory responses executed by IL-6, TNF-α, IL-1β, especially to dead parasites contribute to immunopathology (41).

The proportions of CD8^+^ T cells appear to vary during asymptomatic infections and pathological infections, suggesting that the activities of CD8^+^ T cells in filarial infections are dependent on the stage of the infection. In terms of cellular subsets, individuals presenting with chronic filarial pathologies have elevated levels of activated CD8^+^ T lymphocytes when compared to individuals with Mf only (42). Further analysis of patient sera confirmed the elevation of soluble CD8^+^ T cells in individuals with chronic LF pathology than those at an asymptomatic Mf stage (43). Differential gene expression analysis of blood samples from *Loa loa*-infected individuals originating from Cameroon, Gabon, and Nigeria have defined the functional annotation of gene expression profiles in CD8^+^ T cells (44). The responses to microfilaria antigens by CD8^+^ T cells in endemic individuals were characterized by inflammatory-driven attacks and cell death (44). Furthermore, studies in *Loa loa*-infected rhesus monkeys suggest that CD8^+^ T cells may have a role in the hypo-responsiveness to antigens of *Loa loa* parasites (45). Studies in *B. malayi*-infected monkeys also showed that levels of CD8^+^ T cells were increased in symptomatic models than in asymptomatic ones (46).

CD8^+^ T cells expressing type 1 cytokine responses have been associated with clinical filarial pathologies (2, 31). IFN-γ- and TNF-α-producing CD8^+^ T cells are known to promote filarial pathology particularly through the stimulation of lymphangiogenic growth factors and inflammatory cell migration (47–49). These observations provide preliminary evidence on the involvement of CD8^+^ T cells in filarial pathology. The pathophysiological onset of filarial infections is believed to be driven at the cellular level *via* inappropriate regulation of CD8^+^ T cell activation in an increased pro-inflammatory-mediated fashion (31, 32), as summarized in **Figure 3**.

## Role of CD8^+^ T Cells in Filarial Co-Infection

Helminths are known to be master modulators of host immune responses (50). The immunomodulatory benefits of helminths are being exploited for therapeutic purposes against inflammatory bowel diseases and autoimmunity (51). The filarial immuno-regulatory influence on disease progression and control has been demonstrated with several conditions, including filarial-*plasmodium* co-infection (52), filarial-*mycobacterium* co-infection (53), filarial-virus co-infections (54) and allergic reactions (55).

### Filarial-Virus Co-Infection

Given that both filaria and HIV infections compromise the host immune system, one would anticipate that their co-existence would affect immune responses. It is established that type-1 responses mediate immunity to viruses (56), however, filarial parasites mostly induce a type 2-dependent immunity in their hosts and are capable of regulating these responses to enhance their survival. The suppression of virus-specific CD8^+^ T cell cytotoxicity in HIV-*Schistosoma mansoni* co-infected mice models, with a corresponding delay in viral clearance, has been reported previously (57). The *Schistosoma*-induced reduction in cytotoxic T lymphocyte responsiveness to HIV antigens was markedly due to diminished induction of IFN-γ and IL-2, either through direct IL-10 activities or parasite-induced immunomodulation of innate cells (57). Similar findings in norovirus-*Trichinella* co-infected mice have been reported, where the modulation of viral-specific CD8^+^ T cell responses was observed (58). This inhibition of antiviral immunity was associated with innate immunomodulatory mechanisms during virus-helminth co-infection (58). Thus, the efficiency of viral immune responses can be suppressed during helminth-virus co-infections, at least for *S. mansoni* and *Trichinella spiralis*.

Although the specific immunomodulatory influence filarial parasites have on HIV co-infections *in vivo* is not clearly established, several studies have provided insights into the situation. Nielsen et al. (59) found no influence of HIV-*W. bancrofti* co-infections on the modulation of anti-viral immunity or virus clearance. Notwithstanding, adult *B. malayi* antigen (*Bm*A) influenced HIV-1 trans-infection of CD4^+^ T cells *in vitro* (60). The HIV-1 trans-infection is a common mechanism of virus infection of CD4^+^ T cells by APCs such as dendritic cells and macrophages. The inhibition of CD4^+^ T cell trans-infection involves the blocking of HIV-1 capture and transfer through an interaction between the *Bm*A and dendritic cells, thus, preventing the virus from interacting with dendritic cells (60). The *Bm*A, however, does not influence dendritic cell maturation, cytokine production, and HIV-1 replication in CD4^+^ T-cells (60). Although adult *B. malayi* antigens can influence HIV-1 trans-infection of CD4^+^ T cells, they have no deleterious impact on dendritic cell-derived T helper cytokine profiles against the virus (60). On the role of CD8^+^ T cells in filarial-immunoregulation of viral infections, a considerable gap remains. Dietze et al. (54) studied the immunomodulatory effects of filaria-virus co-infection using an *Litosomodes sigmodontis*-Friend virus (FV) co-infected mice model. Both filaria-specific and viral-specific humoral responses were diminished, but no effect was found on CD8^+^ T cell response to FV infections. While the viral infection did not affect the worm burden, the *L. sigmodontis* infection resulted in increased viral loads due to virus-specific antibody response suppression (54). According to Gopinath et al. (61), the *in vivo* immunological interactions of filaria-HIV co-infections could be properly modeled if cells of individuals with pre-existing filarial infections are used rather than inducing the filarial-immunity with antigens. By infecting CD8^+^ T cell-depleted peripheral blood mononuclear cells from filaria-infected individuals with HIV strains, an indication of increased susceptibility to HIV infections were observed (61).

### Filaria-*Plasmodium* Co-Infection

In addition to their co-endemicity in particular regions, malaria parasites and some filarial parasites share similar transmission vectors. Several studies have explored the possible alterations in host immune responses as a result of filaria-*plasmodium* co-infection (62, 63). Filarial parasites are well known to induce immunosuppressive mechanisms in their human hosts by impairing the production of pro-inflammatory cytokines through the activities of Treg and related cytokines, IL-10 and TGF-β (13, 31, 64). Under the hypothesis that the induced immunosuppression by filarial alters the pro-inflammatory onset of cerebral malaria (CM), Specht et al. (52) studied the development of CM in malaria-infected-murine models with previous filaria (*L. sigmodontis*) infection. CD8^+^ T cells are key players in the progression of CM (65). In the co-infected model, CD8^+^ T cell sequestration into the brain was significantly reduced, which was corroborated with the observed reduction in CM pathology (52). The filaria-induced IL-10 suppresses type-1 cytokines which are known promoters of CM pathologies. In addition, IL-10 mediates the accumulation of CD8^+^ T cells in the spleen, thus, reducing its sequestration into the brain (52). Pre-existing filarial infections in mice models can suppress the production and functioning of CD8^+^ T cells induced by anti-*Plasmodium* vaccination, although the implementation of a heterologous prime/boost immunization regime could stop the observed interference (39).

### Filaria-*Mycobacteria* Co-Infection

Similar to malaria, tuberculosis and filarial infections share a common endemic hotspot. Recent studies have explored the immunoregulatory effect on filaria-*mycobacteria* co-infections (66, 67), however, the precise influence on TB outcome and progression remains open for further investigations. The influence of filarial infections on *Mycobacterium tuberculosis* and *Plasmodium falciparum* infections have been extensively discussed elsewhere (68). Immunity in helminth infections is mostly mediated by Th2 immune responses, whereas immune responses to *M. tuberculosis* require type-1 cytokines (69). CD8^+^ T cells are known inducers of Th1 and Th17 associated cytokines, which are critical components of host immunity against TB (70). It is also well established that filarial parasites can modulate CD8^+^ T cells. The *Mycobacterial* antigen-specific CD8^+^ T cell responses were down-modulated by the occurrence of *W. bancrofti* infection in active pulmonary TB patients (53). Both CD4^+^ and CD8^+^ Th1/Th17 cytokines were diminished in the co-infected mice model, as a result of filaria-induced IL-10 modulation of the pro-inflammatory responses against the *mycobacterium* (53).

### Filarial Infections and Sepsis

In the initial phases of septic attacks, microorganisms mount a robust pro-inflammatory immunity, a stage described as systemic inflammatory response syndrome (SIRS) (71, 72). The compensatory anti-inflammatory response syndrome (CARS) phase immediately follows, which involves an increase in anti-inflammatory molecules and apoptosis of activated immune cells with a corresponding decrease in pro-inflammatory agents (73, 74). This dampens the strength of host adaptive immunity, allowing possible infections with opportunistic microbes (74). Moreover, CD8^+^ T cell cytotoxicity is reduced during the CARS phase (75). Filaria parasites are known to down-modulate CD8^+^ T cell responses and trigger the production of anti-inflammatory cytokines like IL-10 (50). Thus, the alteration of T-cell responses during the SIRS and CARS phases in *Escherichia coli*-induced septic mice model with chronic *L. sigmodontis* infection has been recently explored (76). Interestingly, the weakening of CD8^+^ T cell cytotoxicity through sepsis attack was not promoted by chronic filarial infection (76).

## How Filarial Parasites Could Re-Program CD8^+^ T Cells

The transcriptional factors that regulate CD8^+^ T cell phenotypes and functions are important, particularly during chronic infections (44). How filarial parasites influence the differentiation and subsequent proliferation of naive CD8^+^ T cells is not clear and remain to be established. However, like all other forms of CD8^+^ T cell activation, the presence of a suitable peptide with an MHC class I molecule drives CD8^+^ T cell proliferation and differentiation *via* cross-presentation, given that the filarial parasites are extracellular. An effective CD8^+^ T cell challenge has been implicated in the clearance against viral infections (77) and helminth infections (78) and similar mechanisms could be applied to filarial parasites. Given that filarial pathogens live long in their host, sustained antigen stimulation may alter the differentiation program of CD8^+^ T cells and render them exhausted as previously documented in other infection scenarios (77, 78). These exhausted CD8^+^ T cells are functionally poor since they have reduced lethal impact against their targets. In essence, this could be a possible scenario in filarial infections, where some compromises could be achieved to reduce tissue damage since filarial worms generally survive for at least 10 years in the host. Exhausted CD8^+^ T cells are predominant in chronic infections (77, 79).

Experimental investigation of the functional relevance of regulatory CD8^+^ T cells during infection has advanced, following growth in phenotypic characterization of CD8^+^ T cells subtypes. Activated CD8^+^ T cells release granzyme B, which plays a seminal role during cytotoxic functions. More recently, granzyme B in the regulatory T cell compartment has been found to mediate the suppression of antigen-specific CD8^+^ T cells in viral infection (77). In the same study, increased numbers of antigen-specific CD8^+^ T cells in the lungs of granzyme B-deficient mice were observed, suggesting that granzyme B in Tregs cells regulates either the initiation of antigen-specific CD8^+^ T cells responses or the down-modulation of terminal effector cells or a combination of both. Further, granzyme B has been implicated in enhancing susceptibility during the filarial nematode, *L. sigmodontis* infection (78). Additionally, results from field studies also suggest the suppressive role of granzyme B in human onchocerciasis (79). In this study, the authors observed a high expression of granzyme B and Foxp3 Tregs in the generalized hyporeactive than the hyperreactive individuals (79).

Although the underlying mechanisms dictating CD8^+^ T cell in the presence of filarial antigens is unknown, the fact that increased expression of granzyme B has been associated with immunomodulatory factors such as FoxP3 and TGF-β during filarial infections are good indicators of some immunological cross-talk with CD8^+^ T cell serving as a key player. Thus, we speculate a possible interplay between CD8^+^ T cells and granzyme B during filarial infection in a regulatory fashion among individuals with patent infection. Soluble CD8 (sCD8) released upon activation of CD8^+^ T cells has been documented to reflect filarial disease severity (42, 43).

## The Need for CD8^+^ T Cell Induction by Prophylactic Filarial Vaccines

Currently, the main strategies for controlling filarial infections are through an annual mass drug administration program, management of morbidity, and vector control measures. Despite the progress, millions of individuals are still at risk of filarial infections in over 50 countries worldwide (80). Prophylactic anti-filarial vaccines, replacing or combined with drug therapies, are required to effectively eliminate filarial infections worldwide (81, 82). The search for vaccine candidates for filarial infections has been progressive over the years (reviewed in 81–83), although there are currently no approved consumable vaccines available for filariasis.

Immunization studies in animal models with filarial antigens have provided evidence supporting the efficacy of prophylactic vaccines against filarial infections (84). Several potential vaccine candidates have been identified to offer a wide variety of T cell-mediated responses as part of their protective arsenal against filarial infections in experimental models (85–89). A common observation is that multivalent and cocktail vaccine candidates offer greater protection against filarial infections than single-antigen vaccinations (85–89). This seems plausible, considering the highly complex life-cycle of filarial parasites which involves different stages of development. The majority of immunization trials against filarial infections in experimental models demonstrate induction of IL-2, IL-4, IL-10, IL-17, and IFN-γ, indicating a balanced Th1/Th2 immunity (85–90). The efficacy of these potential vaccine candidates in different animal models ranges from 45% to 94% (83). The vaccine formulations usually include filarial antigens such as heat shock protein 12.6 (86–88), abundant larval transcript-2 (86, 88, 89, 91), tetraspanin large extracellular loop (86–88), vespid allergen homologue (89, 91), thioredoxin peroxide (86), calponin (92), disorganized muscle protein-1 (93), and trehalose-6-phosphate phosphatase (35). In addition to these antigens, adjuvants are added to formulations to improve the vaccine-induced responses. The most used adjuvants for antifilaria immunization formulations are alum, tuftsin and TLR agonists (86, 94–97).

The developmental stages of filarial parasites are complex and are known to regulate several aspects of the cell-mediated immune responses mounted by the host. Emerging evidence shows that CD8^+^ T cells contribute to immune responses against the filaria parasites at the early stages of infection, but are highly regulated to favor parasite survival (31). Given this, a very plausible therapeutic approach would be to prime potential prophylactic vaccine candidates to induce sufficient CD8^+^ T cells and associated memory responses which can have detrimental effects on the establishment of the parasites (15). Identifying CD8^+^ T cell epitopes within filarial antigens could be achieved through immunoinformatics predictions and experimental validations or by screening a library of peptides spanning the complete antigen sequence (83). Recently, our group identified a CD8^+^ T cell peptide (^146^KPWENFMRV^154^) within onchocystatin, as part of a multi-epitope vaccine candidate for onchocerciasis, which demonstrates promising potential based on bioinformatics analysis (98). For most defined T cell antigens, the peptide sequences are usually similar to the corresponding sequences in related filarial parasites which may ensure cross-reaction (98–100).

To accelerate the introduction of filaria vaccines into clinical practice, there is the need to enhance the immunogenicity of potential candidates by boosting their ability to induce CD8^+^ T cell responses. Vaccine development against filarial infections is focused on multi-antigenic or cocktail vaccine formulations, and their cytotoxic mechanisms can be enhanced by conjugating peptides to adjuvants such as TLR ligands (101).

## Concluding Remarks

The current understanding of CD8^+^ T cell-mediated immunity against filarial parasites suggests a type 2-dependent immunity. The CD8^+^ T cell cytotoxic activities appear to be effective in the early stages of filarial infections. Available evidence from animal models and human studies show that CD8^+^ T cells are involved in the immunomodulatory mechanisms that drive the pathogenesis of filarial infections. The mechanistic role of CD8^+^ T cells in filarial pathology could be associated with its poor regulation as a result of filaria-driven suppression, thus, promoting the secretion of pro-inflammatory agents. In the future, the ability to manipulate the CD8^+^ T cell response could have major implications, especially towards developing vaccines against filarial infections. Understanding the dynamics of the CD8^+^ T cells response to infections would help define manipulations that would be optimal for establishing protection against these complex extracellular nematodes. Of note, the recent advances regarding the importance of blocking T cell-derived cytokines or receptors, for instance, provide compelling evidence towards the development of potential therapeutics (102), which could be explored in filarial infections. Here, we have discussed evidence regarding the potential protection of CD8^+^ T cells against filarial infections as well suppression of same during co-infections; thus, suggesting a need for further studies in the future.

## Author Contributions

Conceptualization: AK. Writing - Original Draft: AK, EA, and KK. Writing - Review & Editing: AK, EA, KK, and SA. All authors contributed to the article and approved the submitted version.

## Conflict of Interest

The authors declare that the research was conducted in the absence of any commercial or financial relationships that could be construed as a potential conflict of interest.

## Publisher’s Note

All claims expressed in this article are solely those of the authors and do not necessarily represent those of their affiliated organizations, or those of the publisher, the editors and the reviewers. Any product that may be evaluated in this article, or claim that may be made by its manufacturer, is not guaranteed or endorsed by the publisher.
